# The predictive factors of hypozincemia in patients with chronic liver disease

**DOI:** 10.1017/jns.2025.10062

**Published:** 2025-12-26

**Authors:** Shoji Ando, Atsumasa Komori, Hiroshi Yatsuhashi, Seigo Abiru, Yuri Yotsumoto

**Affiliations:** 1 Department of Nutritional Management, NHO Nagasaki Medical Center, Omura, Nagasaki, Japan; 2 Department of Nutritional Management, NHO Ureshino Medical Center, Ureshino, Saga, Japan; 3 Department of Treatment for Intractable Disease, Clinical Research Center, NHO Nagasaki Medical Center, Omura, Nagasaki, Japan; 4 Clinical Research Center, NHO Nagasaki Medical Center, Omura, Nagasaki, Japan; 5 The Department of Internal Medicine, NHO Saga Hospital, Hinode, Saga, Japan

**Keywords:** Zinc, Chronic liver disease, ALBI score, Albumin, Diuretic, γ-GTP, gamma-glutamyl transpeptidase, ALBI, albumin–bilirubin, ALT, alanine aminotransferase, AST, aspartate aminotransferase, BMI, body mass index, CLD, chronic liver disease, CRP, C-reactive protein, CONUT, controlling nutritional status, FIB, fibrosis, HCC, hepatocellular carcinoma, HOMA-IR, the homeostasis model assessment of insulin resistance, MASLD, metabolic dysfunction-associated steatotic liver disease, MDA, malondialdehyde, NPV, negative predictive value, PPV, positive predictive value, RCTs, randomised controlled trials, ROC, receiver operating characteristic, SOD1, superoxide dismutase 1

## Abstract

Patients with chronic liver disease (CLD) often experience hypozincemia. The clinical factors associated with hypozincemia have not been established. We investigated clinical factors that may be useful to predict hypozincemia in patients with CLD. The serum zinc levels CLD patients were measured; Study 1 investigated the predictive factors of hypozincemia, and Study 2 was performed to validate the factors identified in Study 1. Study 1 included 197 participants, of whom 28 and 106 had serum zinc levels <60 µg/dL and <80 µg/dL, respectively. A multivariate analysis revealed that serum zinc levels <60 µg/dL or <80 µg/dL were associated with the albumin–bilirubin (ALBI) score and serum albumin level. A receiver operating characteristic curve analysis revealed that the ALBI score ≥ −1.83 and the serum albumin level ≤3.3 g/dL were the cut-off values for a serum zinc level <60 µg/dL, whereas the ALBI score ≥ −2.44 and the serum albumin level ≤3.6 g/dL were the cut-off values for a serum zinc level <80 µg/dL. In Study 2 (*n* = 177), the diagnostic accuracy rates for serum zinc <60 µg/dL were 81.9% for the ALBI score and 75.1% for the serum albumin level, and those for serum zinc <80 µg/dL were 70.1% for both parameters. Together these findings indicate that the ALBI score may serve as a predictive factor of hypozincemia in CLD patients.

## Introduction

Zinc deficiency is associated with symptoms such as oral ulcers, increased susceptibility to infections, decreased appetite, dysgeusia, and anaemia.^(1)^ In patients with chronic liver disease (CLD), zinc deficiency is also correlated with disease progression, including liver fibrosis (FIB)^(2)^ and hyperammonemia^(3)^ and is associated with an increased risk of hepatocellular carcinoma (HCC).^(4)^ Clinically, serum zinc levels <60 µg/dL are defined as zinc deficiency, and levels 60–80 µg/dL are defined as marginal zinc deficiency.^(1)^ Patients with CLD often experience zinc deficiency,^(1)^ with a reported prevalence at 28.1%–59.9%^(5,6)^ and even 41.0%–82.8% when restricted to CLD patients with liver cirrhosis.^(5,7,8)^

The factors that contribute to zinc deficiency in persons with CLD are as follows: (*a*) a decrease in albumin-bound zinc due to impaired albumin synthesis in the liver, leading to an increased urinary excretion of amino acid-bound zinc;^(1)^ (*b*) reduced zinc intake due to decreased appetite;^(9)^ (*c*) impaired absorption of zinc from the intestine, possibly due to portal hypertension;^(10,11)^ and (*d)* increased zinc excretion caused by diuretics used for the treatment of ascites and oedema.^(12)^

The literature concerning the need to determine CLD patients’ serum zinc levels (not limited to those with liver cirrhosis) is limited. Even the guidance issued by the America Association of Study of Liver Disease recommends an annual assessment of the serum zinc level in patients with cirrhosis,^(13)^ whereas the European and Japanese guidelines do not clearly state the necessity of evaluating serum zinc.^(14,15)^ Consequently, serum zinc levels are not likely measured routinely in all CLD patients. As such an inadequate assessment can delay the detection and treatment of zinc deficiency in CLD patients, it is important to identify the clinical factors that may be used to predict hypozincemia in order to enable its early detection and timely treatment in at-risk patients, ultimately improving the overall prognosis of individuals with CLD.

Some predictive factors for hypozincemia in patients with CLD have been reported; for example, a serum albumin level ≤3.3 g/dL and a daily furosemide dosage ≥5.0 mg were observed to be associated with serum zinc levels <60 µg/dL.^(16)^ Another study also suggested that the serum aspartate aminotransferase (AST) level, haemoglobin level, and presence of alcoholic liver disease might be potentially useful for predicting hypozincemia in patients with CLD.^(6)^ Although serum zinc levels have been reported to be negatively correlated with the albumin–bilirubin (ALBI) score (which is an indicator of liver function) and the FIB-4 index (a marker of liver FIB)^(2,6,17)^, few studies have evaluated whether these indicators could be used to predict serum zinc levels <60 µg/dL or <80 µg/dL in patients with CLD.

Here we sought to identify predictive factors for serum zinc levels <60 µg/dL and <80 µg/dL in individuals with CLD, with an evaluation of previously described candidate factors, including the ALBI score and FIB-4 index.

## Patients and methods

### Study design

**Figure 1.**
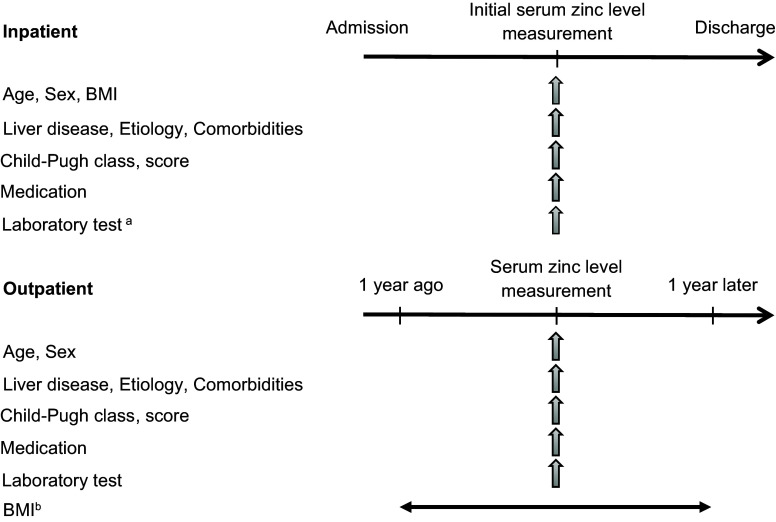
The designs of Study 1 and Study 2, the data items, and the study timeframes. ^a^ Data collected on the same day or within 7 days of the initial serum zinc level measurement. ^b^ BMI collected on the day of serum zinc measurement or, if not available, the most recent value within the prior year. BMI: body mass index.

This study consisted of Study 1 and Study 2, both of which were retrospective cross-sectional cohort investigations. Study 1 was a training cohort in which we analysed the predictive values of the ALBI score and FIB-4 index for serum zinc levels <60 µg/dL and <80 µg/dL. The subsequent Study 2 was performed to validate the predictive factors of hypozincemia identified in Study 1, along with previously reported predictors, i.e., the serum albumin level and the daily furosemide dosage.^(16)^ Figure 1 depicts the study designs.

### Patients

The patients whose cases were eligible for analysis in this study were those with CLD whose morning serum zinc level had been measured and whose ALBI score and FIB-4 index were calculated at the Hepatology Department of National Hospital Organization (NHO) Nagasaki Medical Center. Study 1 included the clinical data of the consecutive inpatients who were examined during the period from April 1, 2020 to March 31, 2022 and the outpatients examined during the period from October 1, 2021 to March 31, 2022. Study 2 was comprised of the inpatients examined during a different period (i.e., April 1, 2018 to March 31, 2020) and the outpatients examined during another period (April 1, 2021 to September 30, 2021).

In order to limit confounders for the patients’ serum zinc levels, we excluded the cases of the patients with any of the following: (*a*) oral zinc preparation use, (*b*) chronic pancreatitis,^(18)^ (*c*) a past history of surgery for pancreatic cancer,^(19)^ (*d*) current participation in haemodialysis or peritoneal dialysis,^(1)^ or (*e*) a past history of gastric, duodenal, or jejunal resection.^(1,20)^ We did not include insufficient zinc intake depending on the patients’ dietary habits as an exclusion criterion, because of both the difficulty of assessment and the retrospective nature of the present analyses. However, our Medical Center applied no dietary restrictions for CLD patients regarding the intake of foods high in zinc, including meat, shellfish, and legumes.

We used all of the above-described exclusion criteria in order to (*i*) minimise the potential influence of factors that could independently affect the serum zinc levels of individuals with CLD, and (*ii*) ensure that all of the variables that are required to test our study hypothesis were available.

### Data collection

The following data were collected during the patients’ hospital admission or at outpatient visits: age, sex, BMI, aetiology of liver disease, comorbidities, Child-Pugh class and score, and the type of medication used. The outpatients’ laboratory data were collected on the same day as the measurement of serum zinc levels, and those of the inpatients were collected within 7 days of the serum zinc level measurements. The outpatients’ BMI was obtained on the same day as the serum zinc measurement, and if it was not available on that day, the most recent BMI within 12 months prior to the serum zinc measurement was used.

The ALBI score was calculated for each patient and classified into three grades: grade 1 (≤ −2.60), grade 2 (> −2.60 and ≤ −1.39), and grade 3 (> −1.39), with a higher score or grade indicating poorer liver function.^(21)^ The FIB-4 index was calculated for each patient and classified into three categories: low (<1.30), intermediate (1.30–2.67), and high (>2.67), with higher values indicating greater liver FIB.^(22)^

### Statistical analyses

The results of the analyses are presented as numbers (%), mean ± standard deviation (SD), or median (interquartile range). Student’s t-test, the Mann–Whitney U-test, the χ^2^-test, and Fisher’s exact test were used to compare the patients with serum zinc levels below and above each of two cut-off values (60 µg/dL and 80 µg/dL). We conducted a binary logistic regression analysis using the backward elimination method with stepwise variable selection to investigate factors that may be associated with serum zinc levels <60 µg/dL or <80 µg/dL. The explanatory variables consisted of (*a*) predefined variables that are likely to have a strong influence on the serum zinc level, including the ALBI score, the FIB-4 index, the serum albumin level,^(1)^ the serum C-reactive protein (CRP) level,^(23)^ the patient’s daily furosemide dosage,^(16)^ alcohol-related aetiology,^(6)^ and the presence of diabetes,^(1)^ and (*b*) other variables that differed significantly between the patients with serum zinc levels below versus above each of the two cut-offs in the univariate analysis.

We performed a receiver operating characteristic (ROC) curve analysis to calculate the cut-off values for the prediction of serum zinc levels <60 µg/dL or <80 µg/dL. The diagnostic accuracy rate was calculated for the validation of the predictive factors for serum zinc levels <60 µg/dL or <80 µg/dL as follows: (true positive patients + true negative patients)/total patients. Probability (*p*)-values <0.05 were accepted as significant. EZR ver. 1.53 software was used for the statistical analyses.

### Ethical considerations

This study complied with the Declaration of Helsinki, and the study protocol was approved by the Ethics Committee of the National Hospital Organization Nagasaki Medical Center (no. 2022081). Consent for participation in the study was obtained through an opt-out approach, with the study details available on the website of NHO Nagasaki Medical Center.

## Results

### Study 1

**Figure 2.**
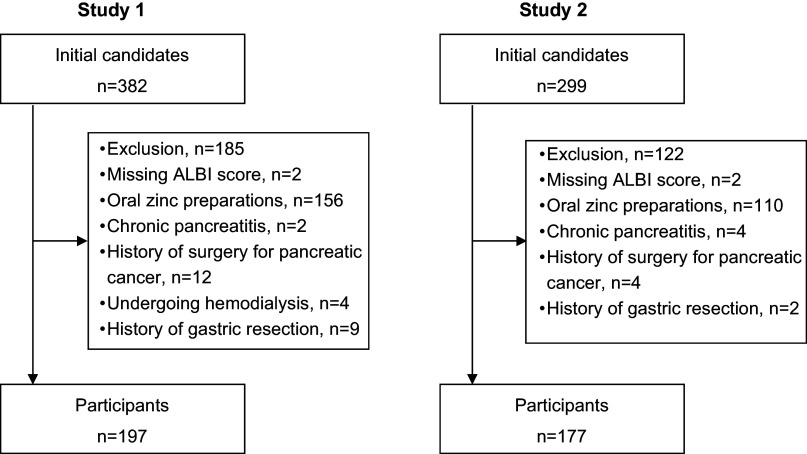
Flowchart of participant selection. ALBI: albumin–bilirubin

Among the 382 initial study candidates of the study, Study 1 analysed the cases of 197 patients, of whom 28 (14.2%) had serum zinc levels <60 µg/dL and 106 (53.8%) had serum zinc levels <80 µg/dL (Figure 2A). Table 1 summarises the patients’ clinical characteristics. A univariate analysis was performed first to identify factors associated with serum zinc levels <60 µg/dL. Briefly (as shown by the data in Table 2), those with serum zinc levels <60 µg/dL had significantly higher ALBI scores (*p* < 0.001), FIB-4 index values (*p* < 0.001), and serum total bilirubin levels (*p* < 0.001), a significantly higher prevalence of diuretic usage (*p* < 0.001), a significantly higher daily dosage of furosemide (*p* < 0.001), and a significantly higher prevalence of liver cirrhosis (*p* < 0.001), as well as significantly lower serum albumin levels (*p* < 0.001).

**Table 1.** Clinical demography of the Study 1 patients (*n* = 197)

Serum zinc, µg/dL	77.2 ± 16.6
Serum zinc <60 µg/dL, n (%)	28 (14.2)
Serum zinc <80 µg/dL, n (%)	106 (53.8)
Inpatient/Outpatient, n (%)	51 (25.9)/146 (74.1)
Age, yrs	68.0 (60.0–77.0)
Male/female, n (%)	108 (54.8)/89 (45.2)
BMI, kg/m^2^	24.8 ± 5.4
Aetiology, n (%):	
HBV/HCV/Alcohol/NAFLD·NASH/ PBC/AIH/Others/Unknown	30 (15.2)/41 (20.8)/41 (20.8)/26 (13.2)/ 9 (4.6)/15 (7.6)/13 (6.6)/22 (11.2)
Liver cirrhosis, n (%)	108 (54.8)
HCC, n (%)	57 (28.9)
History of hepatic encephalopathy, n (%)	21 (10.7)
Ascites and oedema, n (%)	50 (25.4)
Diabetes mellitus, n (%)	73 (37.1)
Child-Pugh	
Class ^a^ A/B/C/Unknown, n (%)	44 (40.7)/34 (31.5)/23 (21.3)/7 (6.5)
Score ^a^	7.0 (5.0–9.0)
ALBI score	− 2.37 (− 2.70 to − 1.83)
Grade 1/2/3, n (%)	68 (34.5)/98 (49.8)/31 (15.7)
FIB-4 Index	3.48 (2.14–5.86)
Cut-off values: low/Intermediate/High, n (%)	19 (9.6)/55 (27.9)/123 (62.4)
Medications:	
Diuretics, n (%)	41 (20.8)
Loop diuretics, n (%)	24 (12.2)
Daily dosage of furosemide, ^b^ mg/day	0.0 (0.0–0.0)
Potassium-sparing diuretic, n (%)	32 (16.2)
Vasopressin V2 receptor antagonist, n (%)	9 (4.6)
Ursodeoxycholic acid, n (%)	34 (17.3)
Proton pump inhibitor, n (%)	79 (40.1)
Laboratory values:	
Albumin, g/dL	3.7 (3.1–4.1)
Total bilirubin, mg/dL	1.0 (0.6–1.8)
AST, U/L	37.0 (25.0–52.0)
ALT, U/L	26.0 (17.0–45.0)
BUN, mg/dL	14.9 (12.1–19.6)
Creatinine, mg/dL	0.79 (0.64–0.96)
CRP, mg/dL	0.71 (0.21–3.05)
Ammonia, mg/dL	68.0 (45.5–95.0)
Prothrombin activation, %	86.3 (73.0–99.9)
WBC,/µL	5,000 (3,800–6,400)
Haemoglobin, g/dL	12.3 ± 2.2
Platelets, 10^4^/µL	14.9 (9.1–20.8)

a Only the patients with liver cirrhosis.

b Daily dosage of furosemide (mg/day): 0, *n* = 173; 10, *n* = 6; 20, *n* = 14; 40, *n* = 3; 60, *n* = 1.

Data are number (%), mean ± SD, or median (interquartile range).

Missing data: BMI, *n* = 29; BUN, *n* = 1; creatinine, *n* = 1; CRP, *n* = 129; ammonia, *n* = 114; prothrombin activation, *n* = 17.

Reference range for laboratory tests; zinc, 65–118 µg/dL; albumin, 4.1–5.1 g/dL; total bilirubin, 0.4–1.5 mg/dL; AST, 13–30 U/L; ALT, 10–42 U/L; BUN, 8.0–20.0 mg/dL; creatinine, 0.46–0.79 mg/dL; CRP, ≤0.14 mg/dL; ammonia, 12–66 µg/dL; prothrombin activation, 70%–130%; white blood cells, 3,300–8,600/µL; haemoglobin, 11.6–14.8 g/dL; platelets, 15.8–34.8 × 10^4^/µL.

AIH: autoimmune hepatitis, ALBI: albumin–bilirubin, ALT: alanine aminotransferase, AST: aspartate aminotransferase, BMI: body mass index, BUN: blood urea nitrogen, CRP: C-reactive protein, FIB-4: fibrosis-4, HBV: hepatitis B virus, HCC: hepatocellular carcinoma, HCV: hepatitis C virus, NAFLD: non-alcoholic fatty liver disease, NASH: non-alcoholic steatohepatitis, PBC: primary biliary cholangitis, WBC: white blood cells.

**Table 2.** Factors associated with serum zinc levels <60 µg/dL

	Univariate analysis				Multivariate analysis			
	Serum zinc < 60 µg/dL *n* = 28	Serum zinc ≥ 60 µg/dL *n* = 169	*p*-value		Analysis of serum zinc levels <60 µg/dL			
					Model 1	*p*-value	Model 2	*p*-value
Serum zinc level, µg/dL	48.8 ± 7.5	81.9 ± 12.5	< 0.001	○1				
Age, yrs	66.0 (53.0–73.0)	69.0 (61.0–77.0)	0.25	○2				
Male, n (%)	20 (71.4)	88 (52.1)	0.089	○3				
BMI, kg/m^2^	24.7 ± 4.2	24.9 ± 5.6	0.88	○1				
Aetiology: Alcohol, n (%)	12 (42.9)	29 (17.2)	0.004	○3	Ref.		Ref.	
Others, n (%)	16 (57.1)	140 (82.8)			–		–	
Liver cirrhosis, n (%)	25 (89.3)	83 (49.1)	< 0.001	○3	–		–	
HCC, n (%)	12 (42.9)	45 (26.6)	0.126	○3				
History of hepatic encephalopathy, n (%)	11 (39.3)	10 (5.9)	< 0.001	○4	–		–	
Ascites and oedema, n (%)	18 (64.3)	32 (18.9)	< 0.001	○3	–		–	
Diabetes mellitus, n (%)	9 (32.1)	64 (37.9)	0.71	○3	–		–	
Child-Pugh class ^a^ A/B/C, n (%)	0 (0)/5 (21)/ 19 (79)	44 (57)/29 (38) / 4 (5)	< 0.001	○3				
Score ^a^	10.5 (10.0–12.3)	6.0 (5.0–7.0)	< 0.001	○2				
ALBI score	− 1.08 (− 1.45 to − 0.85)	− 2.45 (− 2.74 to − 2.16)	< 0.001	○2	19.3 (3.9, 96.2)	< 0.001		
Grade 1/2/3, n (%)	0 (0)/8 (29)/ 20 (71)	68 (40)/90 (53)/ 11 (7)	< 0.001	○3				
FIB-4 Index	7.67 (6.32–9.77)	3.07 (1.97–4.74)	< 0.001	○2	1.2 (1.1, 1.5)	0.012	1.3 (1.1, 1.5)	0.004
Cut-off value: Low/Intermediate/High, n (%)	0 (0)/0 (0)/ 28 (100)	19 (11)/55 (33)/ 95 (56)	< 0.001	○3				
Medication								
Diuretic, n (%)	14 (50.0)	27 (16.0)	< 0.001	○3				
Loop diuretic, n (%)	10 (35.7)	14 (8.3)	< 0.001	○4				
Daily dosage of furosemide, ^b^ mg/day	0.0 (0.0–20.0)	0.0 (0.0–0.0)	< 0.001	○2	–		–	
Potassium-sparing diuretic, n (%)	10 (35.7)	22 (13.0)	0.005	○4				
Vasopressin V2 receptor antagonist, n (%)	2 (7.1)	7 (4.1)	0.62	○4				
Ursodeoxycholic acid, n (%)	5 (17.9)	29 (17.2)	1.00	○4				
Proton pump inhibitor, n (%)	16 (57.1)	63 (37.3)	0.075	○3				
Laboratory values:								
Albumin, g/dL	2.7 (2.4–2.9)	3.8 (3.5–4.2)	< 0.001	○2			0.08 (0.02, 0.3)	< 0.001
Total bilirubin, mg/dL	3.6 (1.8–5.9)	0.9 (0.6–1.4)	< 0.001	○2				
AST, U/L	45.0 (37.8–73.5)	34.0 (23.0–48.0)	< 0.001	○2	–		–	
ALT, U/L	25.0 (17.8–38.3)	27.0 (16.0–46.0)	0.94	○2				
BUN, mg/dL	15.6 (9.4–22.1)	14.8 (12.1–19.1)	0.73	○2				
Creatinine, mg/dL	0.90 (0.78–1.31)	0.77 (0.64–0.92)	0.003	○2	–		–	
CRP, mg/dL	1.92 (0.82–3.59)	0.34 (0.18–1.97)	0.057	○2	–		–	
Ammonia, µg/dL	75.0 (55.0–107.8)	59.0 (41.0–86.0)	0.158	○2				
Prothrombin activation, %	52.1 (40.3–69.5)	88.7 (77.0–101.7)	< 0.001	○2				
WBC,/µL	4,900 (3,180–6,900)	5,000 (3,800–6,300)	0.99	○2				
Haemoglobin, g/dL	10.0 ± 2.4	12.6 ± 2.0	< 0.001	○1	–		–	
Platelets, 10^4^/µL	9.0 (6.1–11.7)	16.2 (9.6–21.3)	< 0.001	○2	–		–	

a Only the patients with liver cirrhosis.

b Daily dosage of furosemide, serum zinc levels <60 µg/dL; 20 mg/day, *n* = 7; 40 mg/day, *n* = 2; 60 mg/day, *n* = 1, serum zinc levels above 60 µg/dL; 10 mg/day, *n* = 6; 20 mg/day, *n* = 7; 40 mg/day, *n* = 1.

○1 Student’s t-test; ○2 Mann–Whitney U-test; ○3 χ^2^-test; ○4 Fisher’s exact test.

Univariate analysis: data are number (%), mean ± SD, or median (interquartile range). Multivariate analysis: values are odds ratio (95% confidence interval).

Multivariate analysis: Of 67 subjects who did not have any missing data for the explanatory variables, 17 had serum zinc levels <60 µg/dL. Binary logistic regression was performed using a stepwise backward elimination method. Excluded explanatory variables were indicated by‘–’. Explanatory variables: The models were divided into Model 1 and Model 2 due to multicollinearity between the ALBI score and the serum albumin levels. The Child-Pugh class and score were excluded as they were only evaluated in patients with liver cirrhosis. The serum total bilirubin levels and prothrombin activity were excluded due to multicollinearity with other variables. The daily dose of furosemide was chosen, due to multicollinearity among the daily dose of furosemide, diuretics, and types of diuretics.

ALBI: albumin–bilirubin, ALT: alanine aminotransferase, AST: aspartate aminotransferase, BMI: body mass index, BUN: blood urea nitrogen, CRP: C-reactive protein, FIB-4: fibrosis-4, HCC: hepatocellular carcinoma, WBC: white blood cells.

In the multivariate analysis, the explanatory variables were analysed separately in Model 1 for the ALBI score and in Model 2 for the serum albumin level, due to these factors’ multicollinearity. We excluded the serum total bilirubin level due to its multicollinearity with the ALBI score.

Consequently, we observed that the ALBI score (odds ratio [OR] 19.3, *p* < 0.001) and FIB-4 index (OR 1.2, *p* = 0.012) were associated with serum zinc levels <60 µg/dL in Model 1. In Model 2, the serum albumin level (OR 0.08, *p* < 0.001) and FIB-4 index (OR 1.3, *p* = 0.004) were significantly associated with serum zinc levels <60 µg/dL (Table 2).

We performed another univariate analysis to identify factors associated with serum zinc levels <80 µg/dL (Table 3). In the multivariate analysis that was conducted with variables that were similar to those in Table 3, explanatory variables were analysed separately in Model 3 for the ALBI score and in Model 4 for both the serum albumin level and the serum total bilirubin level, due to their multicollinearity. In this analysis, the ALBI score (OR 5.2, *p* = 0.002) in Model 3 and the serum albumin level (OR 0.2, *p* = 0.003) in Model 4 were significantly associated with serum zinc levels <80 µg/dL (Table 3).

**Table 3.** Factors associated with serum zinc levels <80 µg/dL

	Univariate analysis				Multivariate analysis			
	Serum zinc < 80 µg/dL *n* = 106	Serum zinc ≥ 80 µg/dL *n* = 91	*p*-value		Analysis of serum zinc levels <80 µg/dL			
					Model 3	*p*-value	Model 4	*p*-value
Serum zinc level, µg/dL	65.0 ± 11.3	91.5 ± 8.4	< 0.001	○1				
Age, yrs	69.0 (61.3–78.0)	66.0 (56.0–74.0)	0.054	○2				
Male, n (%)	67 (63.2)	41 (45.1)	0.016	○3	–		–	
BMI, kg/m^2^	24.6 ± 5.0	25.2 ± 5.8	0.47	○1				
Aetiology: Alcohol, n (%)	26 (24.5)	15 (16.5)	0.23	○3	Ref.		Ref.	
Others, n (%)	80 (75.5)	76 (83.5)			–		–	
Liver cirrhosis, n (%)	71 (67.0)	37 (40.7)	< 0.001	○3	–		–	
HCC, n (%)	38 (35.8)	19 (20.9)	0.031	○3	–		–	
History of hepatic encephalopathy, n (%)	17 (16.0)	4 (4.4)	0.016	○3	–		–	
Ascites and oedema, n (%)	36 (34.0)	14 (15.4)	0.005	○3	–		–	
Diabetes mellitus, n (%)	40 (37.7)	33 (36.3)	0.95	○3	–		–	
Child-Pugh class ^a^ A/B/C, n (%)	20 (29)/25 (37)/ 23 (34)	24 (73)/9 (27) / 0 (0)	< 0.001	○3				
Score ^a^	8.0 (6.0–10.0)	5.0 (5.0–7.0)	< 0.001	○2				
ALBI score	− 2.05 (− 2.43 to − 1.31)	− 2.68 (− 2.99 to − 2.34)	< 0.001	○2	5.2 (1.8, 14.7)	0.002		
Grade 1/2/3, n (%)	15 (14)/61 (58) / 30 (28)	53 (58)/37 (41)/ 1 (1)	< 0.001	○3				
FIB-4 Index	4.19 (2.55–7.29)	2.56 (1.56–4.27)	< 0.001	○2	–		–	
Cut-off values: Low/Intermediate/High, n (%)	3 (3)/24 (23)/ 79 (74)	16 (18)/31 (34)/ 44 (48)	< 0.001	○3				
Medication								
Diuretic, n (%)	29 (27.4)	12 (13.2)	0.023	○3				
Loop diuretic, n (%)	19 (17.9)	5 (5.5)	0.015	○3				
Daily dosage of furosemide ^b^ mg/day	0.0 (0.0–0.0)	0.0 (0.0–0.0)	0.006	○2	–		–	
Potassium-sparing diuretic, n (%)	22 (20.8)	10 (11.0)	0.097	○3				
Vasopressin V2 receptor antagonist, n (%)	8 (7.5)	1 (1.1)	0.040	○4				
Ursodeoxycholic acid, n (%)	21 (19.8)	13 (14.3)	0.40	○3				
Proton pump inhibitor, n (%)	51 (48.1)	28 (30.8)	0.020	○3	–		–	
Laboratory values:								
Albumin, g/dL	3.4 (2.7–3.8)	4.1 (3.7–4.3)	< 0.001	○2			0.2 (0.08, 0.6)	0.003
Total bilirubin, mg/dL	1.1 (0.8–2.2)	0.8 (0.6–1.3)	< 0.001	○2			–	
AST, U/L	39.5 (29.0–53.5)	31.0 (23.0–49.5)	0.023	○2	–		–	
ALT, U/L	25.0 (17.0–42.8)	28.0 (16.5–48.0)	0.53	○2				
BUN, mg/dL	15.5 (12.3–20.9)	14.6 (11.8–18.5)	0.122	○2				
Creatinine, mg/dL	0.84 (0.68–1.06)	0.75 (0.63–0.90)	0.018	○2	–		–	
CRP, mg/dL	0.88 (0.25–3.48)	0.37 (0.18–1.13)	0.27	○2	–		–	
Ammonia, µg/dL	70.5 (47.5–98.8)	58.0 (41.0–77.0)	0.22	○2				
Prothrombin activation, %	78.9 (58.1–91.3)	94.3 (83.5–103.3)	< 0.001	○2	–		–	
WBC,/µL	4,800 (3,700–6,270)	5,200 (4,100–6,400)	0.363	○2				
Haemoglobin, g/dL	11.4 ± 2.1	13.3 ± 1.9	< 0.001	○1	–		–	
Platelets, 10^4^/µL	12.8 (8.6–19.5)	17.3 (11.4–23.5)	0.002	○2	–		–	

a Only the patients with liver cirrhosis.

b Daily dosage of furosemide, serum zinc levels <80 µg/dL; 10 mg/day, *n* = 3; 20 mg/day, *n* = 12; 40 mg/day, *n* = 3; 60 mg/day, *n* = 1, serum zinc levels above 80 µg/dL; 10 mg/day, *n* = 3; 20 mg/day, *n* = 2.

○1 Student’s t-test; ○2 Mann–Whitney U-test; ○3 χ^2^ test; ○4 Fisher’s exact test. Univariate analysis: data are number (%), mean ± standard deviation, or median (interquartile range). Multivariate analysis: values are odds ratio (95% confidence interval). Multivariate analysis: Of the 67 patients who did not have any missing data for the explanatory variables, 48 had serum zinc levels <80 µg/dL. Binary logistic regression was performed using a stepwise backward elimination method. Excluded explanatory variables are indicated by the ‘–’ symbol. Explanatory variables: The models were divided into Model 3 and Model 4 due to multicollinearity between the ALBI score and serum albumin levels and serum total bilirubin levels. Child-Pugh class and score were excluded as they were evaluated only in the patients with liver cirrhosis. The daily dose of furosemide was chosen due to multicollinearity among the daily dose of furosemide, diuretics, and types of diuretics.

ALBI: albumin–bilirubin, ALT: alanine aminotransferase, AST: aspartate aminotransferase, BMI: body mass index, BUN: blood urea nitrogen, CRP: C-reactive protein, FIB-4: fibrosis-4, HCC: hepatocellular carcinoma, WBC: white blood cells.

**Figure 3.**
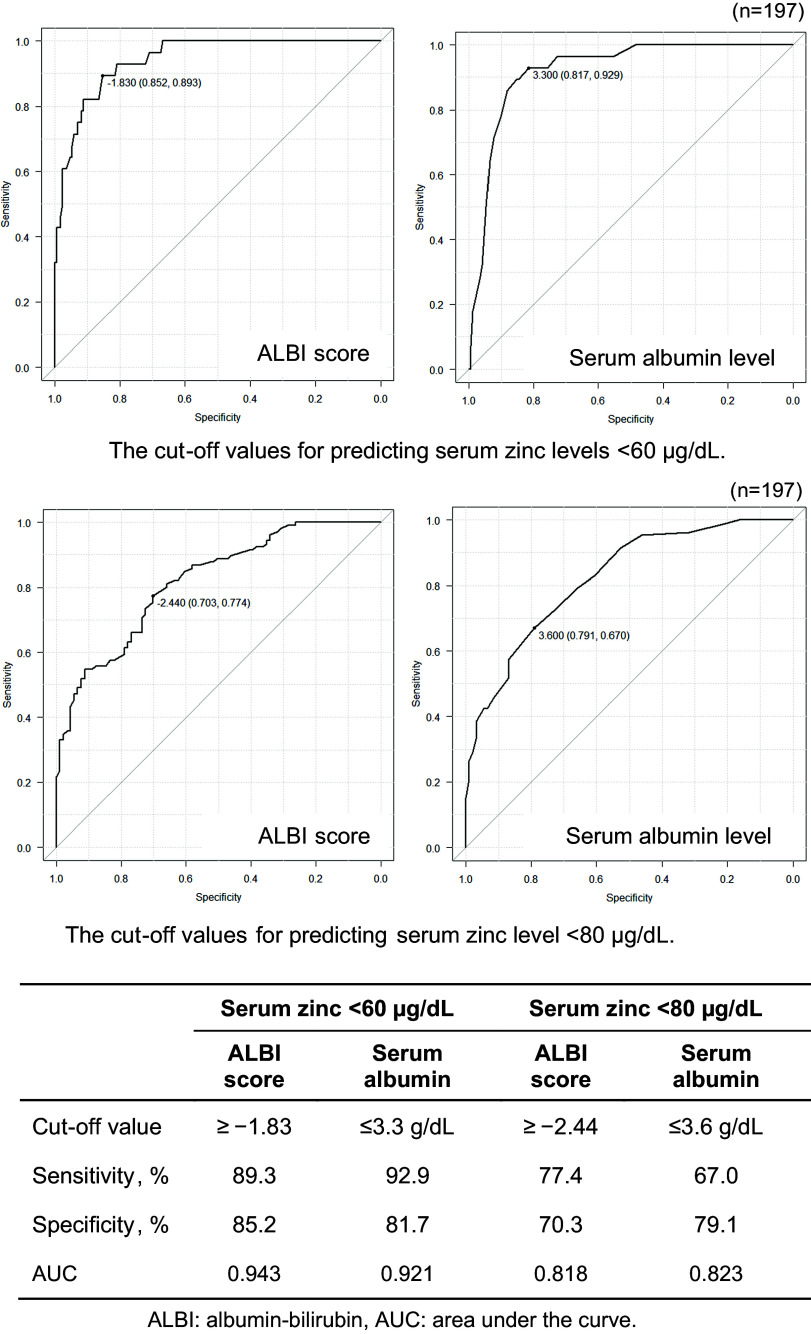
The cut-off values of the ALBI score and serum albumin level for predicting a serum zinc level <60 µg/dL or <80 µg/dL in the ROC curve analysis. The cut-off values were determined as the values at which the sum of sensitivity and specificity is maximised.

Because the ALBI score and the serum albumin level were commonly associated with serum zinc levels <60 and <80 µg/dL, we determined these variables’ cut-off values for each threshold by performing an ROC curve analysis. The results of this analysis demonstrated that an ALBI score ≥ −1.83 and a serum albumin level ≤3.3 g/dL were predictive factors of serum zinc levels <60 µg/dL, and the latter cut-off value for serum albumin (≤3.3 g/dL) was consistent with the finding of an earlier study.^(16)^ An ALBI score ≥ −2.44 and serum albumin level ≤3.6 g/dL were predictive factors of serum zinc levels <80 µg/dL (Figure 3).

### Study 2

Among the 299 initial candidates for Study 2, the cases of 177 patients were analysed, of whom 33 (18.6%) and 92 (52.0%) had serum zinc levels <60 µg/dL and <80 µg/dL, respectively (Figure 2B). Table 4 summarises these patients’ characteristics and shows that the patients’ ALBI scores, serum albumin levels, and daily dosage of furosemide were comparable to those of the Study 1 patients.

**Table 4.** Clinical demography of the Study 2 patients (*n* = 177)

Serum zinc, µg/dL	78.8 ± 19.3
Serum zinc <60 µg/dL, n (%)	33 (18.6)
Serum zinc <80 µg/dL, n (%)	92 (52.0)
Inpatient/outpatient, n (%)	47 (26.5)/130 (73.5)
Age, yrs	69.0 (57.0–74.0)
Male/female, n (%)	101 (57.1)/76 (42.9)
BMI, kg/m	24.2 ± 4.9
Aetiology, n (%): HBV/HCV/Alcohol/NAFLD·NASH/ PBC/AIH/Others/Unknown	25 (14.1)/35 (19.8)/54 (30.5)/24 (13.6)/ 8 (4.5)/8 (4.5)/5 (2.8)/18 (10.2)
Liver cirrhosis, n (%)	106 (59.9)
HCC, n (%)	57 (32.2)
History of hepatic encephalopathy, n (%)	34 (19.2)
Ascites and oedema, n (%)	49 (27.7)
Diabetes mellitus, n (%)	46 (26.0)
Child-Pugh	
Class ^a^ A/B/C/Unknown, n (%)	37 (34.9)/44 (41.5)/20 (18.9)/5 (4.7)
Score ^a^	6.0 (5.0–8.0)
ALBI score:	− 2.30 (− 2.74 to − 1.75)
Grade 1/2/3, n (%)	60 (33.9)/86 (48.6)/31 (17.5)
FIB-4 Index:	3.46 (2.09–5.74)
Cut-off values Low/Intermediate/High, n (%)	23 (13.0)/43 (24.3)/111 (62.7)
Medication:	
Diuretic, n (%)	45 (25.4)
Loop diuretic, n (%)	26 (14.7)
Daily dosage of furosemide,^b^ mg/day	0.0 (0.0–0.0)
Potassium-sparing diuretic, n (%)	34 (19.2)
Vasopressin V2 receptor antagonist, n (%)	13 (7.3)
Ursodeoxycholic acid, n (%)	31 (17.5)
Proton pump inhibitor, n (%)	72 (40.7)
Laboratory values:	
Albumin, g/dL	3.6 (3.1–4.1)
Total bilirubin, mg/dL	1.0 (0.7–1.4)
AST, U/L	36.0 (25.0–51.0)
ALT, U/L	27.0 (16.0–45.0)
BUN, mg/dL	14.7 (11.4–19.4)
Creatinine, mg/dL	0.80 (0.65–0.97)
CRP, mg/dL	0.64 (0.19–2.01)
Ammonia, µg/dL	62.0 (39.0–89.3)
Prothrombin activation, %	84.9 (69.4–99.3)
WBC,/µL	5,100 (3,900–6,800)
Haemoglobin, g/dL	12.3 ± 2.5
Platelets, 10^4^/µL	14.2 (9.3–20.1)

a Only the patients with liver cirrhosis.

b Daily dosage of furosemide 0 mg/day, *n* = 151; 10 mg/day, *n* = 2; 20 mg/day, *n* = 16; 30 mg/day, *n* = 1; 40 mg/day, *n* = 4; 60 mg/day, *n* = 2; 80 mg/day, *n* = 1.

Data are number (%), mean ± SD, or median (interquartile range). Missing data; BMI, *n* = 18; CRP, *n* = 109; ammonia, *n* = 95; prothrombin activation, *n* = 14. Reference range for laboratory tests; zinc, 65–118 µg/dL; albumin, 4.1–5.1 g/dL; total bilirubin, 0.4–1.5 mg/dL; AST, 13–30 U/L; ALT, 10–42 U/L; BUN, 8.0–20.0 mg/dL; creatinine, 0.46–0.79 mg/dL; CRP, ≤0.14 mg/dL; ammonia, 12–66 µg/dL; prothrombin activation, 70%–130%; white blood cells, 3,300–8,600/µL; haemoglobin, 11.6–14.8 g/dL; platelets, 15.8–34.8 × 10^4^/µL. AIH: autoimmune hepatitis, ALBI: albumin–bilirubin, ALT: alanine aminotransferase, AST: aspartate aminotransferase, BMI: body mass index, BUN: blood urea nitrogen, CRP: C-reactive protein, FIB-4: fibrosis-4, HBV: hepatitis B virus, HCC: hepatocellular carcinoma, HCV: hepatitis C virus, NAFLD: non-alcoholic fatty liver disease, NASH: non-alcoholic steatohepatitis, PBC: primary biliary cholangitis, WBC: white blood cells.

We evaluated the diagnostic accuracies of the ALBI score and serum albumin level that had been revealed in Study 1 as well as the previously reported daily dosage of furosemide for the prediction of serum zinc levels <60 µg/dL and <80 µg/dL.^(16)^ The evaluation revealed the following accuracy rates for predicting serum zinc levels <60 µg/dL: ALBI score ≥ −1.83, 81.9%; serum albumin level ≤3.3 g/dL, 75.1%; and daily dosage of furosemide ≥5.0 mg/dL, 79.1%. The accuracy rates of ALBI scores ≥ −2.44 and serum albumin levels ≤3.6 g/dL to predict serum zinc levels <80 µg/dL were both 70.1% (Table 5). Consequently, the ALBI score showed the highest diagnostic accuracy rate for the prediction of serum zinc levels <60 µg/dL or <80 µg/dL.

**Table 5.** Validation for predictive factors of serum zinc levels <60 µg/dL and <80 µg/dL (*n* = 177)

Serum zinc level	Predictive factor	Diagnostic accuracy rate	Sensitivity	Specificity	PPV	NPV
< 60 µg/dL	ALBI score, ≥ − 1.83	81.9	78.8	82.6	51.0	94.4
	Serum albumin level, ≤3.3 g/dL	75.1	84.8	72.9	41.8	95.5
	Daily dosage of furosemide, ≥5.0 mg/day	79.1	33.3	89.6	42.3	85.4
< 80 µg/dL	ALBI score, ≥ − 2.44	70.1	76.1	63.5	69.3	71.1
	Serum albumin level, ≤3.6 g/dL	70.1	69.6	70.6	71.9	68.2

Values are percentages.

ALBI: albumin–bilirubin, PPV: positive predictive value, NPV: negative predictive value.

## Discussion

We investigated and validated factors that can be used to predict hypozincemia in patients with CLD. The main result of our analyses is that the ALBI score could be used to predict hypozincemia among these patients. Other research groups have described a strong correlation between serum zinc levels and the ALBI score (*r* = −0.60 to 0.62, *p* < 0.001), which indicates that the prevalence of hypozincemia increases as the ALBI grade rises.^(6,17)^ Our present findings thus validate and complement previous studies demonstrating the relationship between serum zinc levels and the ALBI score.

The ALBI score was originally developed as an indicator of liver function in patients with HCC, and it has since become increasingly preferred as a measure of liver function in various areas of hepatology. Incorporation of all five components of the gold-standard indicator of the hepatic reserve, i.e., the Child–Pugh score, into a multivariable model revealed that the ALBI score, which examines albumin and bilirubin levels alone in an appropriately derived formula, can be substituted for the Child–Pugh score.^(21)^ Compared to the Child–Pugh score, which is calculated with subjective parameters (presence of ascites and encephalopathy), the ALBI score is able to detect subtle changes in liver function in patients with compensated cirrhosis that cannot be captured by the Child–Pugh score.^(24,25)^ Consequently, the ALBI score has been reported as (*a*) prognostic for mortality in patients with CLD,^(26,27)^ (*b*) predictive of the development of HCC,^(28–30)^ (*c*) prognostic for the development of HCC after treatment,^(31)^ and (*d*) predictive of the complications and outcomes following liver transplantation.^(32,33)^ Beyond liver diseases, the ALBI score has also been suggested to have prognostic utility in patients with heart failure, acute pancreatitis, and various malignancies other than HCC.^(24,34,35)^

Regarding the albumin used to calculate the ALBI score, it is important to note that approx. 60%–80% of the zinc in human blood is bound to serum albumin, and hypoalbuminemia leads to an increase in the urinary excretion of amino acid-bound zinc due to a decrease in protein-bound zinc.^(1)^ Hypoalbuminemia may also contribute to intestinal oedema, potentially reducing the absorption of zinc.^(16)^ Apart from its recently emphasised role as an indicator of inflammation,^(36)^ the serum albumin level remains an important factor in guiding and evaluating nutritional therapy, especially in patients with CLD.^(15)^ The presence of hypoalbuminemia may suggest insufficient nutritional intake and consequently may reflect inadequate zinc intake.

Regarding the other component of the ALBI score, i.e., bilirubin, the details of the mechanisms underlying the association between bilirubin and hypozincemia are unclear. A negative correlation between the serum levels of bilirubin and zinc has been reported.^(37,38)^ Excessive zinc intake may suppress the enterohepatic circulation,^(39)^ whereas zinc deficiency may activate it, potentially compensating for an increased serum bilirubin level.^(37)^ The serum bilirubin level also reflects portal hypertension and hepatic metabolic capacity. As impaired liver function collectively affects zinc intake, absorption, and excretion,^(9–12)^ we suggest that the ALBI score, which includes the serum bilirubin level and can detect subtle changes in liver function, is a more useful predictive factor for hypozincemia than the serum albumin level alone.

In our present investigation, the ALBI score cut-off values for predicting serum zinc levels <60 µg/dL and <80 µg/dL were ≥ −1.83 and ≥ −2.44, respectively, both of which corresponded to ALBI grade 2. The reported prevalence of serum zinc levels <60 µg/dL was 6.7%, 44.6%, and 83.9% in patients with ALBI grades 1, 2, and 3, and the corresponding prevalence of serum zinc levels <80 µg/dL was 74.6%, 92.3%, and 96.8%, respectively.^(17)^ Moreover, another study reported that the prevalence of patients with serum zinc levels <70 µg/dL according to the modified ALBI score — which is considered to provide a more accurate assessment of liver function — was 49.6%, 86.7%, 97.1%, and 100.0% in ALBI grades 1, 2a (> −2.60 and < −2.27), 2b (≥ −2.27 and ≤ −1.39), and 3, respectively.^(6)^ These findings collectively suggest that the prevalence of hypozincemia increases substantially from ALBI grade 2, supporting the validity of the predictive cut-off values identified in our present study.

One possible explanation for the discrepancies between our present findings and the previous reports of a relatively high prevalence of hypozincemia even in ALBI grade 1 is that those studies did not control for the timing of blood sampling, despite the diurnal variation in serum zinc levels (higher in the morning and lower in the afternoon)^(^¹^)^. Our patient population included only patients who had been assessed in the morning, in order to improve the reliability of the results. Though the ALBI score was used as a predictive factor for liver-related mortality, with its cut-off values being relatively high. Our present analyses identified intermediate cut-off values of the ALBI score for the early detection of hypozincemia. The clinical use of these cut-off values may lead to the timely intervention of zinc supplementation, possibly preventing severe hepatic outcomes.

To evaluate the predictive factors for hypozincemia identified in this study, we calculated the diagnostic accuracy rate, which provides a comprehensive assessment of diagnostic performance. We also calculated the sensitivity, specificity, positive predictive value (PPV), and negative predictive value (NPV) to support the interpretation of diagnostic performance. The calculations revealed that the ALBI score provided the highest diagnostic accuracy (81.9%) for predicting serum zinc levels <60 µg/dL, with a favourable balance of sensitivity (78.8%) and specificity (82.6%). Notably, the NPV was as high as 94.4%, suggesting that hypozincemia can be reliably ruled out using the ALBI score. The ALBI score also showed potentially moderate usefulness for predicting serum zinc levels <80 µg/dL, as its predictive performance was inferior compared to that for serum zinc levels <60 µg/dL, warranting further investigation. Taken together, our findings indicate that the ALBI score is a clinically useful factor for predicting hypozincemia, serving as practical guidance for zinc measurement and supplementation.

In the treatment of zinc deficiency, dietary therapy alone is often insufficient, and pharmacological supplementation with 50–150 mg zinc per day in adults is recommended^(^¹^)^. Numerous investigations demonstrated that such supplementation improved patients’ clinical manifestations of zinc deficiency, including oral ulcers, increased susceptibility to infections, dysgeusia, and anaemia.^(40–43)^ Several research groups have reported clinically beneficial effects of zinc supplementation in patients with CLD. Diglio *et al.* conducted a meta-analysis of two randomised controlled trials (RCTs) of 167 patients with CLD to evaluate the efficacy of zinc supplementation for hepatic encephalopathy. Their analysis showed that zinc supplementation significantly improved hepatic encephalopathy (relative risk, 0.66; 95% confidence interval, 0.46, 0.95).^(44)^ Fathi *et al.* investigated 56 patients with metabolic dysfunction-associated steatotic liver disease (MASLD) and obesity, randomised to calorie restriction groups with or without 12-week zinc supplementation. They reported that compared to caloric restriction alone, the added zinc supplementation significantly decreased the patients’ serum alanine aminotransferase (ALT) and gamma-glutamyl transpeptidase (γ-GTP) levels, along with their waist circumference values.^(45)^ In another study conducted by Fathi *et al.* using a similar design, zinc supplementation significantly decreased the patients’ values of serum insulin, the homeostasis model assessment of insulin resistance (HOMA-IR), and oxidative stress markers including serum superoxide dismutase 1 (SOD1) and malondialdehyde (MDA).^(46)^ Hosui *et al.* reported that zinc supplementation improved the liver function of patients with liver cirrhosis and was associated with a reduced risk of HCC development.^(47)^

We also observed that the FIB-4 index was associated with serum zinc levels <60 µg/dL. Hypozincemia has been described as an independent risk factor for liver FIB in patients with MASLD, suggesting a potential link with the FIB-4 index.^(2)^ However, the FIB-4 index is considered a marker of FIB, not liver function,^(48)^ and its calculation does not require knowledge of the patient’s serum albumin level. Because our results did not identify the FIB-4 index as a factor associated with serum zinc levels <80 µg/dL, we consider the FIB-4 index inferior to the ALBI score as a predictive factor of hypozincemia. However, combining the ALBI score with the FIB-4 index has been reported to provide better predictive value for mortality, liver failure, and postoperative recurrence in patients with HCC after hepatic resection.^(48,49)^ It is thus possible that a predictive model incorporating both the ALBI score and the FIB-4 index, such as a regression-based approach, could more efficiently detect hypozincemia. However, predicting hypozincemia by using a regression formula after calculating the ALBI score and FIB-4 index may involve a complicated procedure, and further research is necessary to assess the practicality and generalizability of a predictive model incorporating both the ALBI score and the FIB-4 index.

Our analysis of factors that were previously reported to be associated with hypozincemia (i.e., the daily furosemide dose, serum AST level, haemoglobin level, and alcoholic liver disease), indicated that none of the factors were statistically significant. Furosemide forms a chelate with zinc and also inhibits the reabsorption of zinc in the renal tubules, thereby increasing the urinary excretion of zinc.^(12,16)^ Serum AST levels are thought to be influenced by zinc. In patients with CLD, zinc deficiency may enhance hepatic lipid peroxidation, promote hepatocellular damage, and increase the oxidative stress induced by intracellular iron, leading to elevated serum AST levels.^(50,51)^ In addition, haemoglobin levels are influenced by transcription factors containing zinc fingers, which utilise zinc as a cofactor and play a crucial role in haemoglobin production.^(52)^ Patients with alcoholic liver disease often exhibit zinc deficiency, which is one of the most consistent nutritional/biochemical observations,^(53)^ likely caused by impaired zinc absorption and inadequate intake.^(54,55)^ Although these factors are suspected to be associated with zinc, they likely represent either a consequence of impaired liver function or part of its determinant.; Therefore, in our multivariable analysis, after the adjustment for the effects of the ALBI score and serum albumin levels, these factors were not observed to be significantly associated with hypozincemia.

The Controlling Nutritional Status (CONUT) score, a nutritional prognosis indicator calculated from the serum albumin level, total lymphocyte count, and total cholesterol level, has been proposed as another potential predictor of hypozincemia in patients with CLD. We did not conduct a CONUT analysis in the present study, due to the limited number of patients whose total cholesterol level was evaluated.^(6)^ Patients with zinc deficiency have also been reported to present with clinical symptoms such as lymphopenia, a decreased ratio of T helper (Th) cells to cytotoxic T cells, decreased natural killer (NK) cell activity, and increased monocyte cytotoxicity.^(56)^ In addition, zinc deficiency was demonstrated to affect insulin secretion and insulin resistance, potentially leading to an increased total cholesterol level.^(57)^ It is thus possible that serum zinc levels and the CONUT score are inter-related. However, the correlation between serum zinc levels and the ALBI score has been reported to be stronger than the correlation between serum zinc levels and the CONUT score (*r* = −0.46, *p* < 0.001), suggesting that the ALBI score may be a more sensitive predictor of hypozincemia than the CONUT score.^(6)^

Some study limitations should be addressed. Since the study’s design was retrospective and since the study was conducted at a single institution among patients whose serum zinc levels might have been measured for prophylaxis, selection bias might have occurred. Moreover, 60%–70% of Japanese adults do not meet the recommended dietary intake of zinc,^(58)^ indicating a background of chronic insufficient intake. We did not assess the patients’ zinc intake and thus this variable could not be adjusted for as a potential confounder, which may have influenced the observed associations with serum zinc levels. It thus remains necessary to prospectively analyse consecutive CLD patients in a multicentre study, including the assessment of dietary zinc intake, to avoid such bias and to validate our results.

Second, we analysed the ALBI score as a candidate predictive factor of hypozincemia only among patients with CLD. It remains necessary to evaluate the external validity of our results in populations without CLD but with other diseases. Would the ALBI score be universally predictive of hypozincemia? This is surely an outstanding question in the management of hypozincemia.

## Conclusion

With the use of the ALBI score as a factor for predicting hypozincemia, a patient’s serum zinc level can be measured at appropriate time points as needed, thereby supporting the treatment of zinc deficiency in patients with CLD.

## Data Availability

Data cannot be shared for ethical/privacy reasons.

## Permission to reproduce material from other sources

Not applicable to this study.
